# Successful antibiotic management of Staphylococcus epidermidis endophthalmitis after implantable collamer lens implantation

**DOI:** 10.1186/s12886-023-03127-5

**Published:** 2023-10-12

**Authors:** Ke Zheng, Xiaohong Zheng, Dekang Gan, Xingtao Zhou

**Affiliations:** 1 Department of Ophthalmology, Eye and ENT Hospital, Fudan University, No.19 Baoqing Road, Xuhui District, 200031 Shanghai, China; 2 NHC Key Laboratory of Myopia (Fudan University), Shanghai, China; 3 Laboratory of Myopia, Chinese Academy of Medical Sciences, Shanghai, China; 4 Shanghai Key Laboratory of Visual Impairment and Restoration, Shanghai, China

**Keywords:** Postoperative endophthalmitis, Staphylococcus epidermidis, Implantable collamer lens, Antibiotic therapy

## Abstract

**Purpose:**

We report a case of successful medical management of endophthalmitis post implantable collamer lens (ICL) culture-positive of Staphylococcus epidermidis.

**Observations:**

: A 18-year-old female presented with decreased visual acuity in the left eye 20 days after ICL implantation. A diagnosis of postoperative endophthalmitis was made based on examination and ultrasonography. A vitreous tap was taken, and intravitreal antibiotics (vancomycin 1 mg/0.1ml and ceftazidime 2 mg/0.1ml) were administered twice (every 72 h), and peribulbar injection of triamcinolone acetonide after four days of the second intravitreal injection. The vitreous culture was confirmed for Staphylococcus epidermidis. The endophthalmitis was resolved, and visual acuity improved from 6/20 to 12/20 on day 7 and 22/20 on day 38. This is the first successful medical resolution of Staphylococcus epidermidis endophthalmitis post ICL surgery without ICL explantation and vitrectomy in the V4c model.

**Conclusions and importance:**

: In antibiotic therapy, the excellent compliance and close follow-up of this endophthalmitis patient enabled careful postoperative surveillance on the effect of antibiotic therapy, avoiding the removal of the ICL or the loss of the integrity of the eye. The risk of potential infectious endophthalmitis post-ICL surgery should be fully emphasized during preoperative counseling.

## Introduction

Posterior chamber phakic implantable collamer lens (ICL) implantation was effective and safe for the correction of myopia 1. Infectious endophthalmitis after intraocular surgery is a rare vision-threatening complication. According to the Endophthalmitis Vitrectomy Study, the categories of postoperative infectious endophthalmitis can be divided into acute (occurring within 6 weeks after surgery) or chronic (occurring 6 weeks after surgery) 2. The vitreous tap of most acute cases is confirmed to be Staphylococcus epidermidis and other organisms including gram-negative species. 3 Post-ICL endophthalmitis has been reported previously 4, 5. However, we reported the first successful medical treatment of Staphylococcus epidermidis endophthalmitis without ICL explantation and vitrectomy in the V4c model.

## Case report

A complete vaccination covid-19 test negative 18-year-old female with high myopia and astigmatism and no medical problems came to our clinic to evaluate for refractive surgery. The cycloplegic refraction was − 11.25/-2.50 × 175 OD and − 9.5/-2.00 × 170 OS. The best corrected distance visual acuity (BCVA) was 20/20 in both eyes. The intraocular pressure (IOP) was 18.9 and 19.3 mmHg. The endothelial cell density was 3245 cells/mm^2^ OD and 3190 cells/mm^2^ OS. The white-to-white diameter was 12.1 and 12.3 mm, and the anterior chamber depth (from endothelium) was 3.38 and 3.49 mm, respectively. The central corneal thickness was 541 μm and 535 μm, the mean K-value was 43.7D and 44.2D. In view of high refractive error, a decision for bilateral ICL-V4c implantation was made. Informed consent was obtained after explaining the potential risks of surgery. V4c TICL (VTICMO13.7–15.0/+2.5 × 113 OD VTICMO13.7–13.0/+2.5 × 113 OS) (Visian, STAAR Surgical Co, California, USA) were implanted through the temporal incision under topical anaesthesia, after ophthalmic viscosurgical device (OVD) was administered over the ICL, the ICL was adjusted using the manipulator, the haptics tip was positioned on the ciliary sulcus and the viscoelastic agent was replaced with a balanced salt solution. The incisions were sutureless closed with a balanced salt solution, the surgery was uneventful. Topical Levofloxacin 0.5% eyedrops four times a day and prednisolone acetate 1% eyedrops four times a day were started immediately after the surgery. The uncorrected distance visual acuity (UDVA) was 20/20 in both eyes on the first postoperative day; there was no corneal edema, and 1 + cells were present in the anterior chamber (AC). The ICLs were in situ with a vault of 660 μm OD and 550 μm OS on pentacam.

**Fig. 1:**
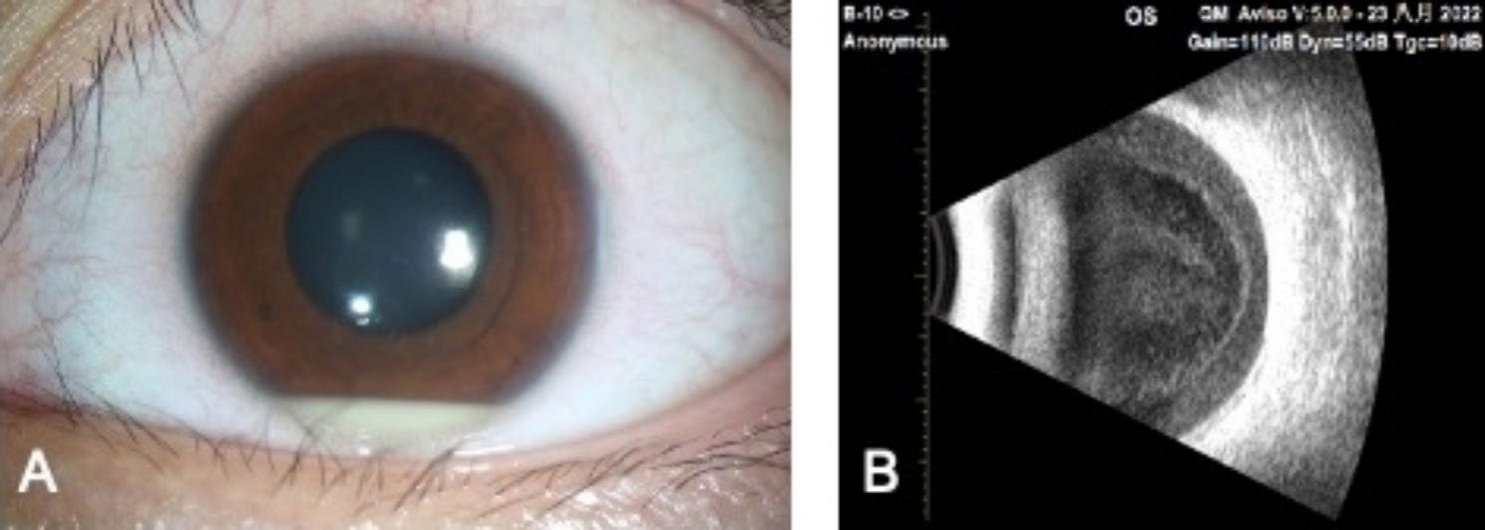
(**A**) An anterior segment examination on postoperative 20 days showed 1 mm hypopyon and fibrin exudate; (**B**) Longitudinal B-scan demonstrated vitreous opacities

The patient presented with blurred vision and redness of the left eye 20 days following bilateral ICL surgery. She was noted to have participated in an intensive bicycle racing competition hosted in Shanghai 3 days ago. UDVA was 20/20 OD and 10/20 OS with IOP of 21.8 mmHg and 15.9 mmHg, respectively. Slit lamp exam showed clinical manifestations, including mild conjunctival and ciliary congestion, white round keratic precipitates, 3 + anterior chamber flare, and cells, with fibrin exudate, 1 mm hypopyon in the anterior chamber, the ICL was in a good position. Vitreous opacity OS; Dilated fundus exam was invisible (Fig. 1A and B). The right eye was unremarkable.

## Treatment

Considered for postoperative endophthalmitis OS, a vitreous tap and intravitreal injection of vancomycin 1 mg/0.1ml and ceftazidime 2 mg/0.1ml was performed. The sample was sent for pathogen detection and drug sensitivity testing. The patient was also treated with systemic (intravenous administration of Ceftazidime every 24 h) and topical antibiotics (Tobramycin-dexamethasone eyedrops every 15 min, Levofloxacin 0.5% eyedrops every 2 h), topical prednisolone acetate 1% eyedrops three times a day and topical tropicamide 0.5% eyedrops once a day.

**Fig. 2:**
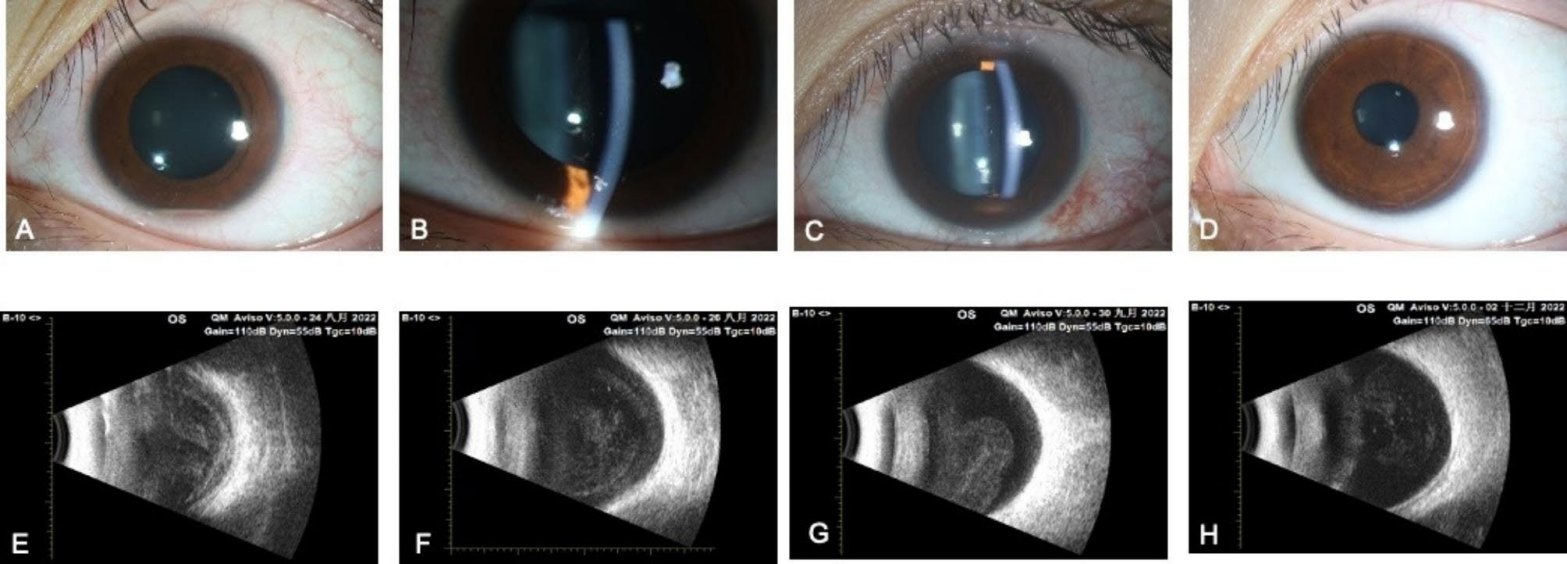
(**A, E**) 24 h after the first intravitreal injection slit lamp examination showing fibrin exudate, 0.5 mm hypopyon in the anterior chamber. Longitudinal B-scan shows the presence of multiple mild to moderate amplitude echoes in the vitreous cavity;(**B, F**) 72 h after the first intravitreal injection, anterior segment examination showed inferior keratic precipitates. Longitudinal B-scan shows persistent vitreous opacities;(**C, G**) Complete resolution of inflammation in the anterior chamber 38 days post-antibiotic therapy. Longitudinal B-scan showing the gradual resolution of the vitreous opacity; (**D, H**) 3 months post antibiotic therapy the anterior chamber and B-scan was unremarkable

The cultures were positive for Staphylococcus epidermidis, which was sensitive to all antibiotics. The patient was diagnosed with postoperative endophthalmitis. 24 h after intravitreal injection, the BCVA was 6/20, with the resolution of hypopyon. (Fig. 2. A and E). Systematic antibiotic treatment was maintained (intravenous administration of Ceftazidime) over the subsequent three days.

Given persistent inferior keratic precipitates, anterior chamber flare and cells, and vitreous opacity, UDVA remained 6/20, fibrin exudation in the pupil area and the vitreous body was much less (Fig. 2.B and F), intravitreal injection of vancomycin 1 mg/0.1ml and ceftazidime 2 mg/0.1ml was performed 72 h again later. As the AC inflammation and ICL deposits were minimal, we did not remove the ICL nor AC washout, and a close follow-up was arranged to monitor the progress of endophthalmitis. After four days of the second intravitreal injection, the UDVA was 12/20, anterior chamber flare and cells (+) remained, and peribulbar injection of triamcinolone acetonide 20 mg/0.5ml was administered, with the aim of anti-inflammatory effect of corticosteroid injections to improve visual acuity and reduce the inflammation in the vitreous in a short time.

### Outcome and follow up

The topical treatment was tapered gradually over one month. There was a notable improvement in AC reaction, complete resolution of exudates, and gradual resolution of the vitreous opacity, BCVA improved to 22/20 with inactive vitreous opacities 38days post the antibiotic therapy (Fig. 2C and G). The UDVA of 20/20 was achieved 3 months following the antibiotic therapy, the manifest refraction was − 0.25/-0.50 × 65 = 20/20, the anterior chamber was unremarkable, persistent vitreous opacities were resolved (Fig. 2D and H).

## Discussion

ICLs are ciliary sulcus placed anterior to the natural lens to treat myopia, correcting refractive error ranging from − 0.5D to -18D. It is an alternative, especially for patients with abnormal corneal topography and high myopia, and can provide better postoperative visual quality in patients with high myopia in the early postoperative period 6. Infectious endophthalmitis is one of the most vision-threatening complications post-ICL surgery. The rate of endophthalmitis post-ICL patients was approximately 1 in 6000(0.017%~0.036%) [7]. Cases of endophthalmitis caused by Staphylococcus epidermidis 5, Pseudomonas aeruginosa 8, and Cutibacterium acnes 9, 10 postoperative have been reported, which required removal of the ICL during the treatment and replanted (Table 1). The signs and visual outcomes vary among different causative microorganism and onset of presentation. In acute postoperative endophthalmitis Streptococcus species and gram-negative organisms are more common, and in chronic postoperative endophthalmitis Cutibacterium acnes (formerly Propionibacterium acnes) are more common. Patients with acute endophthalmitis generally presented with poor initial acuities and visual outcomes than chronic endophthalmitis.

**Table 1:** Comparisons of published case reports of endophthalmitis post ICL implantation

Report (year)	Nation	Etiology	Onset	Organisms	Initial VA	VAN	Treatment	Final VA
Davis MJ (2009) [4]	USA	ICL (V4b)	4d	coagulase-negative Staphylococcus epidermidis	20/40	S	IVAB	20/20
Oum BS (2011) [8]	Korea	PPC IOL (exchange)	1 m	Pseudomonas aeruginosa	LP	S	IVAB, PPC IOL removal, PPV with a lensectomy, IOL implantation in the sulcus after 6 months	20/30
Kaur M (2015) [5]	India	ICL	24 h	methicillin-resistant Staphylococcus epidermidis	HM	S	IVAB, ICL explanted and repeat implantation after 9months	20/20
Robbins CC (2021) [10]	USA	ICL	5 m	Cutibacterium acnes	N/A	S	IVAB, ICL removal and lensectomy, PPV	20/20 (aphakia)
Wilkinson S (2022) [9]	USA	ICL	3 m	Cutibacterium acnes	20/30 − 1	S	IVAB, ICL and native lens removal, PPV, IOL was placed in the sulcus after several months	20/20

PPC IOL,posterior chamber intraocular lens;ICL,implantable collamer lens;LP,light perception;HM,hand movement;VAN,vancomycin;S,susceptible; IVAB,intravitreal antibiotics; PPV,pars plana vitrectomy;h,hour(s);d,day(s);m,month(s);N/A,not available;

Virulence of the pathogens is considered a critical factor in the prognosis. The inflammation is often slowly progressive for chronic postoperative endophthalmitis, since it is commonly caused by less virulent bacteria. Poor prognosis cases mostly require intraocular lens removal, capsulectomy, radical vitrectomy with hyaloid peeling, base dissection, and silicone oil 8, 11, 12. Host factors that lower resistance to infection, such as chronic immunosuppressive therapy and diabetes mellitus, have also been reported to be significant risk factors for postoperative endophthalmitis 13. The major source of infection is the patient’s resident ocular surface or lid skin flora, intraocular isolates were found identical to simultaneous lid skin isolates in several cases 14. In our case, the early postoperative recovery was uneventful, the temporal self-sealing clear corneal incision may be the portal of entry for the Staphylococcus epidermidis 15. There is another case report of culture-positive coagulase-negative Staphylococcus epidermidis without ICL removal 4. The model of ICL, in this case, is V4b without a central hole (Table 1). Our case is the first successful antibiotic treatment of Staphylococcus epidermidis endophthalmitis without ICL explantation or vitrectomy in model V4c with a central hole and two additional holes facilitating the aqueous outflow.

Test the virulence and antibiotic susceptibilities and resistance traits of Staphylococcus epidermidis strains causing postoperative endophthalmitis may offer more helpful information in the treatment selection and prognostic evaluation in a clinical setting 16.To our knowledge, Staphylococcus epidermidis isolates were sensitive to vancomycin 12. ICL explantation would be required in cases of methicillin-resistant Staphylococcus epidermidis 5. In our case, intraocular vancomycin and ceftazidime was used for empiric coverage of gram-positive and gram-negative organisms in the primary procedure, the Staphylococcus epidermidis endophthalmitis post ICL responded well to intravitreal antibiotics, avoiding the necessity of pars plana vitrectomy.

Slit lamp evaluation is critical in diagnosing, predicting the causative organisms, and guiding the therapeutic decisions before the microbiologic confirmation. In our case, the treatment started with intravitreal injection and system administration of antibiotics accompanied by intensive topical steroids and antibiotics before the culture confirmation. An ultrasonography B-scan can assist in the diagnosis. The close follow-up of this endophthalmitis patient enables strict postoperative surveillance on the effect of medication treatment, avoiding the removal of the ICL or pars plana vitrectomy.

## Conclusion

To conclude, Staphylococcus epidermidis endophthalmitis is manageable and can be recovered without loss of vision when diagnosed and treated in time and correctly. Good patient compliance plays an essential role in successful management of Staphylococcus epidermidis endophthalmitis post ICL avoiding the loss of the eye’s integrity. The risk for endophthalmitis is rare but should be fully emphasized during preoperative counseling.

## Data Availability

Available upon request from Dr. Ke Zheng.
